# Meteorological Table

**Published:** 1813-04

**Authors:** 


					356
METEOROLOGICAL TABLE.
From February 25, to March 25, IS 13.
?
o
51 46'ao1
43 41 [SO2
52 40
51 4S|?
48 44 304
47 43[30*
54 47
52 43
44 43
39 31
39 33
34 36
39 43
50 4?
51 45
54 45
53 50
52 44|29s
48 43
53 51
55 44
51 45
47 45I301
48 43|302
Quantity of rain from the 25th of February to the 25th of March,
of an inch.
The character of this interval has been an unusual calmness, with a
small quantity of rain, and generally a high temperature. For a few days
toward the beginning of March, the weather severe; and, in some po-
sitions, the mercury in the thermometer sunk to 29: where this register is
kept, however, it was never below 31. At this time snow fell in greater
quantity iii London that at any previous time during the winter.
Inflammations in the chest, rheumatic fevers, and affections of the
bowels, resembling the autumnal cholera, have frequently occurred.
MONTHLY

				

